# Postpartum Depression: Current Status and Possible Identification Using Biomarkers

**DOI:** 10.3389/fpsyt.2021.620371

**Published:** 2021-06-11

**Authors:** Yi Yu, Hong-Feng Liang, Jing Chen, Zhi-Bin Li, Yu-Shuai Han, Jia-Xi Chen, Ji-Cheng Li

**Affiliations:** ^1^Central Laboratory, Yangjiang People's Hospital, Yangjiang, China; ^2^Center for Analyses and Measurements, College of Chemical Engineering, Zhejiang University of Technology, Hangzhou, China; ^3^Institute of Cell Biology, Zhejiang University, Hangzhou, China

**Keywords:** postpartum depression, postnatal depression, biological markers, biomarkers, multi-omics

## Abstract

Postpartum depression (PPD) is a serious health issue that can affect about 15% of the female population within after giving birth. It often conveys significant negative consequences to the offsprings. The symptoms and risk factors are somewhat similar to those found in non-postpartum depression. The main difference resides in the fact that PPD is triggered by postpartum specific factors, including especially biological changes in the hormone levels. Patients are usually diagnosed using a questionnaire onsite or in a clinic. Treatment of PPD often involves psychotherapy and antidepressant medications. In recent years, there have been more researches on the identification of biological markers for PPD. In this review, we will focus on the current research status of PPD, with an emphasis on the recent progress made on the identification of PPD biomarkers.

## Introduction

Postpartum depression (PPD) has raised a major public health concern. It has been estimated that about 15% of women within 1 year after childbirth may suffer from PPD (1). Like major depression, PPD is a disabling disorder. Significant negative effects of PPD on children, even after they grow into adulthood, have been documented in the literature. PPD-related suicide has become the second-leading cause of death for women in the postpartum period (2). While antidepressants have been effective in treating PPD in many cases, possible side effects of antidepressant medication have been of great concern. Therefore, it is crucial to identify PPD at an early stage.

Although, known since 400 BC, PPD it did not catch wide attention until about half a century ago. Over the past decades, there have been many research efforts and reviews in the field of PPD. Grace et al. (3) reviewed the literature and found that PPD confers small effects on cognitive development such as language and IQ. Behavioral effects may persist up to 5 years post partum and beyond. Dennis and McQueen (4) reported that women with depressive symptoms at the early stage of the postpartum period were associated with increased risk for negative infant feeding outcomes. The review by Blum (5) focused on the psychodynamics of PPD, and found that a triad of three common, specific emotional conflicts (dependency conflicts, anger conflicts, and motherhood conflicts) was typical of many women who develop PPD. Yawn et al. (6) found that among those evaluated programs between 2000 and 2010, only four studies included patient outcomes, and only two reported success in improving outcomes. O'Hara and McCabe (7) found that the results of most studies did not seem to converge, and the majority had a small sample size or suffered from a lack of proper controls. Anderson and Maes (8) reviewed the biological aspects of PPD, and suggested that tryptophan catabolites, indoleamine 2,3-dioxygenase, serotonin, and autoimmunity play a powerful role in immuno-inflammation and oxidative and nitrosative stress. Furthermore, decreased level of endogenous anti-inflammatory compounds together with decreased ω-3 poly-unsaturated fatty acids (PUFA) in the post-partum period may be a central cause for PPD. Kim et al. (9) conducted a review on the role of oxytocin in the treatment of PPD, and found that the results was inconsistent. Yim et al. (10) conducted a review on researches published between 2000 and 2013 on the predictors for PPD and found that the biological and psychosocial literatures were largely disconnected, and integrative analyses were rare to find. They reported that the strongest biological predictors for PPD risk were hypothalamic-pituitary-adrenal (HPA) axis dysregulation, inflammatory processes, and genetic vulnerabilities, while the strongest psychosocial predictors were severe life events, chronic strain, poor relationship quality, and family support. There are many other reviews on PPD in the literature, which can be classified mainly into two distinct categories: biological vs. psychosocial approaches. The former addressed the endocrine system, the immune system, and genetic factors (11–14), while the latter addressed stressors and interpersonal relationships (5, 15–17). Reviews that covered both categories (18, 19) are relatively rare.

In the present review, we shall emphasize on the literature on PPD in the past several years. There has been an increasing number of researches on the screening and diagnosis of PPD using biological markers. Several biomarkers have been identified using the modern technology of multi-omics. The omics-based biomarkers can provide a more quantitative and objective criterion for the diagnosis of PPD, compared with the questionnaire-based diagnosis.

## Diagnosis of PPD

There remains a controversy regarding the criterion for the onset time of PPD (1). The Diagnostic and Statistical Manual of Mental Disorders revision 5 (DSM-5) of the US encompasses episodes that sets in during pregnancy (20) and that begin within 6 months of delivery. In clinical practice and in various studies in the literature, the onset time for PPD has been generalized to up to 1 year post-partum.

A clinical interview can be used to diagnose PPD, such as the Structured Clinical Interview for the DSM-IV (21). Alternatively, easy-to-use self-report measures such as questionnaires have also been widely used for clinical assessment. The most prominent and widely used is the Edinburgh Postnatal Depression Scale (EPDS) (22), which has been reported to be reliable, well-validated, and often more practical and cost-effective in wide-scale screenings for PPD risk (23). EPDS lays an emphasis on psychic symptoms of depression, so as to reduce the weight of common symptoms of most new mothers. Other commonly used questionnaire-based screening tools include two-item Patient Health Questionnaire (PHQ-2) (24) and 9-item Patient Health Questionnaire (PHQ-9) (25). PHQ-2 contains the first two items of the PHQ-9. A typical EPDS or PHQ-9 score of 10 or above is used as the cutoff for being PPD positive. Brief subscales of EPDS have also been designed (26), such as 3-item, 7-item and 2-item subscales. Other screening tools include the Hamilton Rating Scale for Depredession (HAM-D) (27), which was not designed for PPD specifically. The reliability of HAM-D varies significantly in different evaluations, ranging from 0.46 to 0.98 (28). Other scales for diagnosing related mood disorders, such as the Bipolar Spectrum Diagnostic Scale (BSDS) (29) for bipolar disorders (BD), may also become relevant when such disorders occur during the perinatal period.

## Etiological Models of PPD

The exact causes of PPD are still unknown. Models of PPD can mainly be divided into two categories: biological vs. psychological models. Integrated models of both are rare. However, many existing studies suffer from relatively small sample sizes or lack of control (or both), and none of the models mentioned here is conclusive.

### Biological Models

It is well-known that childbirth is accompanied by a dramatic decreases in several hormones, such as estradiol, progesterone, and cortisol. In **withdrawal models**, reproductive hormones (30) and stress hormones (31) rise dramatically during pregnancy and then drop suddenly upon delivery, and thus leads to system dysregulation and hence PPD (32). These models cannot explain how the hormonal withdrawal acts to cause depression in women, nor can they explain those depressive symptoms that begin during pregnancy before parturition.

In **depression models**, PPD is associated with dysregulation of *stress hormones*, particularly cortisol (12). Several recent reviews suggested that dysregulation of the HPA axis plays a main role in the development of PPD (33, 34). Diminished dopaminergic function may also play a role in PPD (35). Sudden estradiol withdrawal could lead to dysregulation in brain dopaminergic pathways and hence PPD. Multiple neuroendocrine changes caused by pregnancy may also play a role in PPD development, including dysfunctional gamma-aminobutyric acid (GABA) signaling (36, 37). PPD has also been found to be associated with low allopregnanolone levels during pregnancy (38). The involvement of GABA and allopregnanolone in PPD has also been hypothesized in other models of PPD pathophysiology (39).

### Psychological Models

Psychological models emphasize the deleterious role of psychological stressors and underlying cognitive vulnerabilities and the ameliorating role of psychosocial resources. In these theories, pregnancy, childbirth, and new parenthood are stressors that cause women to develop PPD symptoms. One can find consistent support for these models in the psychological literature (32, 40, 41).

### Integrated Models

An integrated model may bridge the biological and psychological theories. For example, in the stress vulnerability model, stress can cause PPD symptoms in women that have genetic, hormonal, and cognitive vulnerabilities (32). The bio-psycho-social-cultural model of Halbreich (42) is a combination of the stress vulnerability model with biological and cultural factors. There exists limited evidence in the literature that supports these integrated models.

### Evolutionary Models

Models from an evolutionary perspective regard PPD as a consequence of modern civilization, due to psychological adaptation during the human evolution. Hagen (43) proposed that PPD could cause parents to reduce or eliminate investment in infants that may have health and development problems, and it also may help them negotiate greater levels of investment from others. Recently, Hahn-Holbrook and Haselton (44) proposed a “mismatch hypothesis” of PPD that dramatic cultural changes that have occurred over the past century leads to significant divergences from the typical lifestyles throughout human evolutionary history, and gives rise to the current high incidence rate of PPD, suggesting that PPD may be a “disease of civilization.”

## Treatment of PPD

Psychological treatment usually happens in the form of counseling (or psychotherapy), either one-on-one with a psychologist or in a group setting (45), and has been extremely beneficial for many women. Some women can effectively recover from their depression through counseling alone, while others may have to undergo counseling in conjunction with the use of antidepressants. Thus far, strong support can be found in the literature that a variety of psychological treatments of PPD can be effective.

Medical treatment for PPD includes pharmacotherapy with antidepressants. In fact, antidepressant medication is the most common treatment for PPD (46). There have been extensive investigations of a broad spectrum of antidepressants in the treatment of PPD, and many have been found to be associated with symptomatic improvement (46), and to be as effective as usual care plus counseling (47). Recently, an allopregnanolone-based treatment for PPD, brexanolone, now commercially called Zulresso®, has been approved by the Food and Drug Administration (FDA) in the United States as a fast-acting, long-lasting antidepressant (48). In double-blind, randomized, controlled clinical studies, brexanolone injection has been observed to be associated with a rapid reduction at 60 h in depressive symptoms compared to placebo, and the reduction could sustain no <30 days with broad responses (49).

Regarding the drug exposure of the infant through breastfeeding, a few reviews (50–53) concluded that nortriptyline, paroxetine, and sertraline have the strongest safety performance during lactation.

It should be noted that PPD may share some common genetic and biological risk factors and biomarkers with other mood disorders such as the major depressive disorder (MDD) and the bipolar disorder (BD). In this sense, PPD may be viewed as a forming part of a unique mood spectrum (54, 55). Accordingly, these common features may cause misdiagnosis. Indeed, patients with BD may sometimes be misdiagnosed as having MDD, leading to inappropriate treatment with antidepressant medication (29, 56). The misuse of antidepressants in patients with BD without mood-regulators may induce (hypo)mania or rapid cycling and may increase the risk of illness recurrence (57–60). Therefore, to ensure proper treatment, extra caution must be excised to ensure proper diagnosis.

## Psychosocial Risks Factors for PPD

A very large body of literature has addressed risk factors for PPD based on cross-sectional and prospective studies (45). According to several meta-analyses of risk factors for PPD (15, 16), risk factors can be categorized based on the strength of their association with PPD. Depression and anxiety during pregnancy, postpartum blues, history of depression, neuroticism, stressful life events, poor marital relationship, and poor social support, low self-esteem, as well as some cognitive emotion regulation strategies (61) have been found to have a strong or moderately strong association. A large-scale population based study (62), using data on more than 700,000 deliveries in Sweden between 1997 and 2008, reported that the risk of PPD for women with a depression history was over 20 times higher than women without. On the other hand, low socioeconomic status (SES), single marital status, unwanted pregnancy, obstetrical stressors, and difficult infant temperament (63, 64) have been reported to exhibit a relatively weaker association. Maternal attitudes (65), women's experience of a various related complications such preterm birth, prenatal hospitalization, emergency cesarean section, pre-eclampsia, and poor infant health (66), can also cause an elevated risk of developing PPD (67–69). These risk factors are more closely related to the social and psychological aspects rather than biological aspects.

## Biological Predictors and Biomarkers for PPD

In recent years, more development has been made to identify the biological predictors for PPD. Substantial biological changes can be associated with pregnancy. Such changes are necessary in order to maintain normal pregnancy and fetal development, as well as successful labor and lactation. Upon parturition, the intricate balance that has developed during gestation to sustain the maternal-placental-fetal unit is suddenly no longer needed. Furthermore, the maternal system has to undergo a dramatic biological changes into the lactation phase within a short time. It may days or even months to re-establish a new biological balance. It is conceivable that failure to re-establish the balance properly and promptly may cause maternal mental health issues in return.

### Genetic and Epigenetic Studies

It is generally hoped that investment of possible genetic causes of a psychiatric disorder may help to reveal the underlying pathophysiological mechanism, which can help to find cures or improved treatments. Studies revealed that there may exist an underlying genetic cause for PPD (70). Viktorin et al. (71) found that the heritability of perinatal depression was estimated at 54 and 44%, respectively, in twin and sibling samples, which means that about half of the variability in perinatal depression can be explained by genetic factors. This is substantially higher than the heritability of non-perinatal depression at 32%. Forty et al. (72) and Murphy-Eberenz et al. (73) reported that PPD with onset within 4 weeks post-parturm exhibits familiality in families with MDD. These studies suggest that, while it also has its own unique features, the genetic basis for PPD may partially overlap with that for other mood disorders.

Unlike MDD, there have been relatively fewer studies addressing genetic contribution to PPD. A partial summary of genetic association studies of PPD was tabulated by Payne (70). In these studies, various genes were investigated for their roles in PPD symptoms, such as those associated with the regulation of the HPA axis, sex hormones, and the effects of stress on the prefrontal cortex (1). Mahon et al. (74) examined the genetic etiology of postpartum mood disorders using genome-wide data. They found that genetic variations on chromosomes 1 and 9 may increase susceptibility to postpartum mood symptoms, for women who had a history of pregnancy and any best-estimate mood disorder diagnosis. Specifically, the genes HMCN1 and METTL13 may contain polymorphisms that confer susceptibility to postpartum mood symptoms. Nevertheless, these associations were not significant enough to sustain statistical corrections from multiple testing. Alvim-Soares et al. (75) found that a HMCN1 polymorphism (rs2891230) is associated with PPD symptoms, and the heterozygosity for this single nucleotide polymorphism (SNP) was associated with an increased risk of PPD, in a sample of 110 randomly selected, unrelated Brazilian women of European descent, assessed at 8 weeks postpartum. Their result seems to support the finding of Mahon et al., however, future studies with a larger sample size are certainly needed. Costas et al. (76) reported a significant association (with *p* = 0.002) between the SNP rs11924390 between SNP at the transcriptional start site of kininogen 1 and PPD during the first 32 weeks after delivery. Clear signatures of gene expression of mononuclear cells were found in woman with PPD symptoms as compared with healthy controls (77, 78). The usefulness of this study, however, suffered from its small sample size. Further studies with a larger sample size are warranted in order to confirm these associations. Nonetheless, while these results differ, they are not inconsistent with one another.

The serotonin transporter gene (SER T) has been one of the most widely studied candidate gene associated with PPD (70). It has two primary polymorphisms, 5-HTTLPR and STin2VNTR (10). The former contains a 44-bp deletion or insertion in the promoter region, corresponding to the short and long allele variants, respectively. The latter polymorphism involves a variable number of tandem repeats (VNTR) in the second intron, among which the longer VNTRs have been found to be associated with mental health issues and depressive disorders. So far, studies have shown mixed results on the role of SER T polymorphisms in PPD. Lesch and Mössner (79) found that the short allele may be associated with higher risk of developing PPD. However, the long allele variant of the 5-HTTLPR (serotonin transporter, i.e., 5-HTT, linked polymorphic region) was also reported to be associated with PPD symptoms at 6 weeks (80, 81) or within 1 year post-partum (82).

A weak link between estrogen receptor gene (ESR1) and PPD has been reported (76, 83), which, however, failed to remain statistically significant when corrections for multiple tests were taken into account. It was also suggested that the role for ESR1 in the etiology of PPD could possibly be mediated through the modulation of serotonin signaling (83). Mehta et al. (84) found that women with PPD (with onset within 7 weeks after delivery) displayed an increased sensitivity to estrogen signaling in comparison with controls. While these studies indeed support the idea that estrogen plays a role in the development of PPD, they are far from being conclusive, partly because of the relatively small sample size in these studies.

Catechol-Omethyltransferase (COMT) and monoamine oxidase-A (MAO-A) are allelic gene variations in the monoaminergic system. They have been shown to be related to MDD. The former is involved in dopamine and noradrenalin metabolism, while the latter plays a role in the degradation of serotonin and noradrenaline in the brain (85). A few studies revealed that MAO-A and COMT may be associated with PPDs that have an onset within 8 weeks postpartum (75, 81, 86). Sacher et al. (87) found an association between PPD and greater MAO-A V_T_ (an index of MAO-A density) in the prefrontal and anterior cingulate cortex, compared to healthy controls.

Oxytocin has been found to plays an important role in physiological and genetic systems that permit the evolution of the human nervous system and allow the expression of contemporary human sociality, and stress reactivity. It acts to allow the facilitation of birth, lactation, and maternal behavior (88, 89). Decrease in the oxytocin level in plasma has been associated with PPD (90, 91). Possible roles in PPD have also been investigated for polymorphisms of the oxytocin receptor (OXTR) gene, the oxytocin peptide gene (92), the glucocorticoid receptor gene and the CRH receptor 1 gene (93). However, only some weak, suggestive links have been reported. Jonas et al. (94) found that polymorphisms in OXT rs2740210 interacted with early life adversity to predict PPD. Bell et al. (95) found that for women who do not show depression during pregnancy, but possess the rs53576_GG genotype and exhibit high levels of methylation in OXTR, the risk of developing PPD was nearly three times that for women of lower methylation levels. Kimmel et al. (96) found that the cytosine-guanines (CpGs) located on chr3 at positions 8810078 and 8810069 were associated with PPD scores significantly for a cohort of 240 women without a psychiatric history. They also found a PPD specific negative correlation between DNA methylation in the region and serum estradiol levels. In addition, estradiol levels and OXTR DNA methylation exhibited a significant interaction to associate with the ratio of allopregnanolone (ALLO) to progesterone. Recently, King et al. (97) found that mothers with persistent perinatal depression (depressive symptoms both prenatally and postpartum) exhibited significantly higher overall OXTR methylation at 16/22 individual CpG sites. While these studies seem to support the link between (the epigenetic DNA methylation of) OXTA and the risk of developing PPD, it should be noted that these findings are mostly derived from small sample sizes, with variable depression rating scales and a lack of prospective measures of DNA methylation, and thus should be interpreted with caution.

The brain-derived neurotrophic factor (BDNF) system is known to play an important role in many neuronal functions (15). Serum BDNF was found to decline considerably across pregnancy from 1st through 3rd trimesters (*p* ≤ 0.008) and subsequently to increase at postpartum (*p* < 0.001), and lower serum BDNF in late pregnancy was reported to be associated with higher depressive symptoms (98), although low BDNF levels were found to persist even 2 months after birth (99). Aydemir et al. (100) and Gazal et al. (101) found that the BDNF levels in serum of PPD patients were lower than in healthy control subjects. Serum BDNF levels in PPD patients that had a suicide risk were significantly lower than those of women that did not show a suicide risk (102). Recently, Fung et al. (103) found an association between lower levels of serum BDNF in early pregnancy and antepartum depression. However, Figueira et al. (104) and Comasco et al. (80) did not find an association between PPD and the BDNF polymorphism Val66Met. Overall, clear evidence for the association between BDNF and PPD symptoms is yet to be found (10). This may be partly due to the fact that the normal serum level of BDNF is a non-monotonic function of time in the perinatal period. Thus the precising timing and its dynamics may be important. The lack of consensus in the literature may partly reflect the difference in experiment design in terms of timing and sampling strategy, besides the often small sample sizes.

Katz et al. (105) collected maternal RNA longitudinally from preconception through the third trimester of pregnancy in 106 women with a lifetime history of mood or anxiety disorders. They reported that mRNA expression of a number of glucocorticoid receptor (GR)-complex regulating genes was up-regulated over pregnancy, and women with depressive symptoms showed significantly smaller increases in mRNA expression of four of these genes. They also found that GR sensitivity diminished with increasing maternal depressive symptoms. A prospective pregnancy cohort study of 56 healthy women with singleton term pregnancies found that altered placental genes expression involved in glucocorticoid and serotonin transfer may function as potential gestational-age-specific marker of PPD risk (106). Further studies with a larger sample size are needed to replicate these findings.

Recently, by RNA sequencing the whole transcriptomes of peripheral blood mononuclear cells, Pan et al. (107) found that PPD was positively correlated with multiple genes involved in energy metabolism, neurodegenerative diseases and immune response, while negatively correlated with multiple genes in mismatch repair and cancer-related pathways. In addition, genes associated with appetite regulation and nutrient response were differentially expressed between PPD (*n* = 56) and control subjects (*n* = 27).

**Epigenetics** refers to changes in gene function that do not alter the DNA sequence itself. The main focus in this area has been mostly on DNA methylation, which can be modified by medication and stress, as well as reproductive hormones. There has been some progress in the identification of biomarkers for DNA methylation. Recent studies have implicated epigenetic processes in the pathophysiology of MDD (108). Guintivano et al. (109) and Kaminsky and Payne (110) observed enhanced sensitivity to estrogen-based DNA methylation reprogramming in those at risk for PPD and identified two potential biomarker loci at the HP1BP3 and TTC9B genes that predicted PPD. Using blood drawn during pregnancy, DNA methylation at two genomic locations with an area under the receiver operator characteristic (ROC) curve [area under the curve (AUC)] of 0.87 in antenatally euthymic women and 0.12 in a replication sample of antenatally depressed women, along with complete blood count data, produced an AUC of 0.96 across both prepartum depressed and euthymic women (109). Osborne et al. (111) found that TTC9B and HP1BP3 DNA methylation in early antenatal stage showed moderate association with the change in estradiol and ALLO levels over the course of pregnancy, suggesting that epigenetic variation at these loci may be important for mediating hormonal sensitivity, and that PPD is mediated by differential gene expression and epigenetic sensitivity to pregnancy hormones and thus modeling proxies of this sensitivity may enable accurate prediction of PPD. Osborne et al. (38) further found an association between lower ALLO levels in the second trimester of pregnancy and an elevated risk of developing PPD, which seem to have been confirmed by latest studies (39, 49, 112–114), and can be traced back to the two genes identified above. Indeed, this seems to have gathered more support than many other biomarkers. Very recently, Payne et al. (115) found that antenatal TTC9B and HP1BP3 DNA methylation may be used to predict both antenatal and postpartum depression.

A prospective study (109) of 93 pregnant women with a history of either MDD or bipolar disorder found significant correlation between PPD risk and 17β-estradiol (E2)-induced DNA methylation change, suggesting that an enhanced sensitivity to estrogen-based DNA methylation reprogramming exists in women at risk for PPD. Estradiol increases the rate of transcription of the OXTR gene (116), resulting in elevations of oxytocin levels in the uterus (117) and in numerous brain regions (118) while heterozygosity for the OXTR rs2254298 polymorphism can interact with early life adversity to yield the highest levels of symptoms of depression, physical anxiety, and social anxiety (119). Association between higher DNA methylation of the OXTR gene and decreased expression of the gene was also observed (120). A case control study (95) on the OXTR gene DNA methylation at CpG site-934 and genotype rs53576 and rs2254298 found that women with GG genotype had higher risk of developing PPD with increasing methylation level. The finding of King et al. (97) regarding OXTR methylation seem to suggest that the onset timing of PPD is also an important factor.

Overall, the above findings suggest that polymorphic variations in candidate genes within the monoaminergic system can have an effect on the estrogen receptor, the oxytocin peptide, the glucocorticoid receptor, and the CRH receptor 1 genes, and may act as potential biomarkers for PPD. However, further investigation is needed in order to determine whether the short or long allele of the 5-HTTLPR is associated with PPD risk and under what conditions. Nevertheless, the lack of consensus in these data from the literature highlights the complex relationship between epigenetics and PPD related neuroendocrine changes.

### Reproductive hormones

The association between the reproductive hormones and PPD has been studied and reviewed, in terms of estrogens, progesterone, prolactin, oxytocin, and testosterone. Bloch et al. (121) found evidence that the reproductive hormones estrogen and progesterone play a role in the development of PPD. However, the data by Klier et al. (122) did not support the hypothesis of a role of sex hormones in the etiology of PPD. A review of about 200 studies by Serati et al. (123) found little evidence that supports estrogen withdrawal theories, or suggests that progesterone in late pregnancy or postpartum period predicts PPD symptoms.

Recent studies seem to suggest strong association between the progesterone level and PPD. The levels of ALLO was found to increase progressively throughout gestation, and drop rapidly upon parturition (37, 124, 125). As a metabolite of progesterone, ALLO is a neuroactive steroid measurable in peripheral circulation. Therefore, its levels vary proportionally with progesterone levels throughout gestation and after the delivery, and an association between ALLO and PPD were found, suggesting that hormonal regulation plays an important role in the development of PPD (124). Previously, Bloch et al. (121) found that women with a PPD history are more sensitive to mood-destabilizing effects of gonadal steroids than healthy controls. Rather than progesterone withdrawal upon delivery, it has been found that a low ALLO level during pregnancy predicts PPD (38, 112, 114). Such an association was also found in earlier studies (126, 127). Timing may be an important factor when assessing potential biomarkers. Epperson et al. (128) reported that cortex GABA levels and plasma ALLO concentrations were reduced in two different groups of postpartum women, regardless of PPD diagnosis at 9 weeks or 6 months postpartum, compared to healthy follicular phase women, and that no correlation was found between cortical GABA concentrations and estradiol, progesterone, or ALLO levels. Smith et al. (129) found that the effect of neuroactive steroids on inhibition, which influence anxiety state and seizure susceptibility, depends not only on the subunit composition of the receptor but also on the direction of Cl^−^ current generated by these target receptors.

Prolactin has physiological functions that are especially relevant in the peripartum period, and may act as an attenuation for behavioral and neuroendocrine stress responses during both pregnancy and lactation (130). Despite mixed evidence from different studies, two studies with a big sample size indeed suggested an negative correlation between PPD and prolactin (131, 132).

The oxytocin signaling network has been of great interest as it can play an important role in mother-infant bonding and interactions. Recently, there have been investigations on the trajectories of oxytocin throughout the gestation and lactation periods, especially how they respond to the onset and development of PPD symptoms. It has been suggested that lower levels of oxytocin in both the gestation and postpartum periods may imply an elevated risk for developing PPD (9, 90, 91). Jobst et al. (133) found that plasma oxytocin levels significantly increased from week 35 of gestation to 6 months postpartum in all women. However, levels decreased from the 38th week of gestation to 2 days after delivery in women with PPD, whereas, they increased continuously in the healthy control group. This suggests that the time evolution pattern of oxytocin may be a predictor of PPD in the immediate postpartum period (within 2 weeks). In comparison, Massey et al. (134) found that oxytocin level interacted with past MDD to predict PPD symptom severity in the third trimester; a higher oxytocin level predicted greater PPD symptom severity in women with past MDD, but not in women without. Thul et al. (135) reviewed the literature on the relationship between both endogenous and synthetic oxytocin and PPD, and found that out of the 12 studies that focused on endogenous oxytocin, eight studies suggested an inverse correlation between plasma oxytocin levels and depressive symptoms.

There is mixed evidence for the association between testosterone levels in late pregnancy and PPD symptoms in the early postpartum stage. Women with PPD symptoms were reported to have higher serum testosterone levels in the late third trimester (136) and around 24 h postpartum than healthy controls (137). However, other studies have failed to find such an association (138).

The role of thyroid hormone in the development of perinatal mood disorder has also been investigated (139). It has been suggested that timing may be critical in the correlation between thyroid hormones and PPD, since the function of thyroid is to respond to the constant changes in other hormones across gestation. Kuijpens et al. (140) reported that the presence of thyroperoxidase antibody (TPOAb) during gestation was associated with the occurrence of subsequent depression during the postpartum period and as such can be regarded as a marker for depression. Elevations in thyroid stimulating hormone (TSH) upon delivery have been proposed to be a predictor for PPD 6 month post-parturm (141). However, in an earlier large cohort study, Albacar et al. (142) examined 1,053 postpartum Spanish women without a previous history of depression, and concluded that thyroid function at 48 h after delivery does not predict PPD susceptibility. Groer and Vaughan (143) found that pregnant TPO-positive women were more likely to develop PPD 6 months after delivery. A review (144) suggested that TPOAb in early to mid-pregnancy was associated with concurrent depression and may be predictive of PPD. Recently, Wesseloo et al. (145) found that women with an increased TPOAb titer during early gestation were at increased risk for self-reported first-onset depression, which suggested an overlap in the etiology of first-onset PPD and autoimmune thyroid dysfunction. Li et al. (146) found that PPD patients showed elevated serum levels of triiodothyronine, thyroxine, free triiodothyronine, free thyroxine along with diminished estradiol, progesterone, and TSH levels. It was proposed that it may not just be thyroid hormones alone, but rather thyroid in conjunction with other factors such as estrogens (10) or trauma history (147), that are implicated in PPD etiology. A consensus regarding the role of thyroid hormones as a biomarker is yet to be reached.

While strong evidence seems to have been found to support that a low ALLO level during pregnancy predicts PPD, it can be seen, however, that for most reproductive hormones, a consensus regarding their association with PPD is yet to be reached. Many studies are limited by small sample sizes. Furthermore, the dynamical changes of reproductive hormones during the perinatal period should also be taken into account. This will require that the timing for sampling and depression assessment be arranged in a more systematic and consistent manner, hopefully across different studies.

### Stress Hormones

Stress hormones, particularly those of the HPA axis, have been implicated in non-puerperal depression (148). It was suggested that the hyporesponsiveness of the HPA axis may persist for several months postpartum (149). There is strong evidence that the HPA axis plays an important role in perinatal depression, both during gestation and postpartum. Groer and Morgan (131) reported that depressed mothers had a down-regulated HPA axis, in that the salivary cortisol level was lower in PPD patients than in healthy controls. Similar to reproductive hormones, the levels of stress hormones also increase during pregnancy from the first to third trimester and then decrease abruptly upon parturition. In contrast, the neuropeptide corticotropin-releasing hormone (CRH) often increases exponentially in the process of pregnancy (150), mainly because CRH is also produced by the placenta (151). This also leads to an increased level of adrenocorticotropic hormone (ACTH) and cortisol over the course of pregnancy (152). The CRH level drops quickly upon parturition, when the placenta is discharged. Yim et al. (153) found that at a critical period in midpregnancy, placental CRH (pCRH) is a sensitive and specific early diagnostic test for PPD; at 25 weeks' GA, pCRH was a strong predictor of PPD, and the trajectories of pCRH in women with PPD are significantly accelerated from 23 to 26 weeks' GA. Hahn-Holbrook et al. (154) found that steeper increases in placental CRH from 29 to 37 weeks' gestation predicted more depressive symptoms postpartum. Iliadis et al. (155) reported an association between high CRH levels in gestational week 17 and the development of PPD symptoms, among women without depressive symptoms during pregnancy. On the contrary, Meltzer-Brody et al. (34) found that higher mid-pregnancy placental CRH was *not* associated with an increased risk of PPD. Glynn and Sandman (156) showed that depressive symptoms at 3 months postpartum were associated with elevated mid-gestational pCRH levels and also accelerated trajectories of pCRH, but pCRH was not predictive of PPD at 6 months postpartum. They concluded that elevated pCRH level during pregnancy may act as a marker of risk of developing PPD. Overall, the result on CRH as a viable biomarker is mixed so far, possibly due to limitations of small sample sizes and different timing for sampling and assessment of the PPD symptoms.

Alteration in the HPA axis is a robust biomarker of anxiety and depression, and significant mood symptoms in pregnancy was shown to be associated with altered diurnal cortisol in pregnancy (157). Labad et al. (158) found that women with postpartum thoughts of harming the infant had higher ACTH levels, when compared to those women without intrusive thoughts, and a dysregulation of the HPA axis may play a role in the etiology of postpartum thoughts of harming the infant. Jolley et al. (159) found higher ACTH and lower cortisol levels in women with PPD at 6 and 12 weeks postpartum when compared with controls. Women with PPD were found to have greater ACTH stress reactivity to cold pressor test (CPT), and a significantly elevated ACTH concentration level at 8 weeks postpartum in response to CPT (160), as well as a markedly blunted plasma ACTH response to serial ovine CRH tests at 3, 6, and 12 weeks postpartum (161), and it was suggested that the suppressed ACTH response to ovine CRH might serve as a biochemical marker of the postpartum “blues” or depression (161). Together, these findings again suggested that dysregulation of the HPA axis may be associated with PPD. However, a comparative study found no differences in the HPA axis reactivity in terms of the cortisol and ACTH response, e.g., the cortisol/ACTH ratio, to pharmacologic test and psychological challenges during the luteal phase between current euthymic postpartum women with a history of either PPD or MDD and controls (162). We note that the sample sizes for the last four studies were fairly small, with 12, 34, 17, and 15 × 3 (15 in each group), respectively. Further investigations with larger sample sizes and better designs are needed to reconcile these findings.

As for β-endorphin, Yim et al. (163) found that among women who were euthymic at 25 weeks' GA, those who developed PPD had higher β-endorphin levels throughout pregnancy than women without PPD symptoms, and suggested that β-endorphin may play an important role in the pathophysiology of PPD and may thus be a useful early predictor of PPD symptoms in women who show no depressive symptoms in mid-pregnancy. They also reported that around 25 weeks' gestation was a crucial time for assessing PPD symptoms. However, postpartum blood samples were taken only at 9 weeks for assessment of β-endorphin levels, and self-report was used to evaluate the depressive symptoms.

Parcells (164) found that cortisol levels directly correlated with maternal depression, anxiety, and stress. Recent studies found that women with PPD had higher salivary evening cortisol at 6 weeks postpartum (165), elevated hair cortisol levels in the first to third trimesters (166), or lower levels of evening cortisol in the immediate peripartum period (167), compared to healthy controls. Corwin et al. (168) found that cortisol levels, together with family history of depression and interleukin (IL)-8/IL-10 ratio, were significant predictors of PPD symptoms. However, a recent survey (169) showed that most studies reported no association between maternal cortisol and antenatal depression, and that among studies that reported an association, second-trimester and third-trimester cortisol assessments more consistently reported an association. The link between cortisol levels and postpartum or perinatal depressions is far from conclusive, and thus more future investigations are warranted.

Among stress hormones, it is less controversial that alteration of the HPA axis is a robust biomarker for PPD. However, results on CRH, ACTH and cortisol levels are rather mixed, and further studies are needed on these hormones as well as β-endorphin.

### Immunological/Inflammatory Studies

The function of the immune system is to protect the body from foreign substances (as well as other pathogenic organisms) (10). However, the fetus should not be attacked during pregnancy even though it is necessarily genetically distinct and carries paternal antigens that are foreign to the maternal immune system. Thus this constitutes a big challenge for the maternal immune system. Although, it is not yet clear how it maintains the proper balance of proinflammatory cytokines [e.g., IL-6, IL-1β, tumor necrosis factor-alpha (TNF-α)] and anti-inflammatory cytokines (e.g., IL-10), it can be expected that the immune system will have more activities during the perinatal period. A growing body of literature suggests that inflammatory responses have an important role in the pathophysiology of depression (170). In addition, prolonged HPA axis hyperactivity activated by proinflammatory cytokines (IL-1, IL-6, and TNF-α) is one of the mechanisms underlying cytokine-induced depression (171), even in perinatal episodes (172).

It has been found that puerperal women usually have significantly higher levels of proinflammatory cytokines during the last trimester of pregnancy, and are also at higher risk for depression (173). Other stressors also cause proinflammatory cytokine levels to rise at such a time. Breastfeeding may attenuate stress and modulate the inflammatory response. Overall, the proinflammatory state during the late pregnancy and the early postpartum period plays an important role in the development of PPD (173). IL-6 is the most commonly studied cytokine, and has been consistently identified as being elevated in depression. An earlier study (174) found that the levels of serum IL-6 and its receptor (IL-6R) were significantly higher in the early puerperium than before delivery, and women who developed depressive symptoms in the early puerperium had significantly higher serum IL-6 and IL-6R concentrations than those without. Corwin et al. (175) reported an increase in IL-1β on Day 14 and Day 28 postpartum in women with PPD, compared to levels in euthymic women, suggesting an association between symptoms of PPD and elevated levels of IL-1β during the first month postpartum. Boufidou et al. (176) found that cytokine IL-6 and TNF-α levels in the cerebrospinal fluid (CSF) and TNF-α levels in serum were positively associated with depressive mood during the first 4 days postpartum and also at sixth week postpartum. Krause et al. (177) found that regulatory T cells in pregnancy strongly predicted PPD. Corwin et al. (168) found that family history of depression and cortisol AUC and the IL8/IL10 ratio both on day 14 were significant predictors of PPD. Liu et al. (178) found that elevated serum IL-6 at delivery was associated with development of PPD during the 6 months post partum. In a longitudinal study (179), the IL-6 and IL-10 levels measured in the third trimester were found to be a significant predictor of PPD. However, the *evidence is mixed* for the link between PPD and proinflammatory cytokines (10). For example, serum leptin levels at delivery were found earlier (180) to be negatively associated with self-reported depression during the first 6 months after delivery, but were significantly greater in women with PPD than in healthy controls at 3 months post-partum (181). However, a similar association was not found between serum IL-6 levels at delivery and later PPD symptoms.

Other proinflammatory cytokines (and their receptors) have also been investigated, as potential biomarkers for PPD. Groer and Morgan (131) found that women with PPD had lower serum levels of Interferon-gamma (IFN-γ) and a lower IFN-γ/IL-10 ratio in both serum and in whole blood stimulated cultures. Fransson et al. (182) found that mothers with depression had higher TGF-β2 concentrations in their breast milk than mothers without depression. They also found associations between maternal IL-6, IL-8 and cord IL-6, IL-8, IL-10, IL-13, and IL-18 levels and depressive symptoms in the first 5 days postpartum in women who delivered preterm. Clara Cell Protein (CC16), an endogenous anticytokine, may also be related to PPD. Maes et al. (183) found that parturients who developed PPD had significantly lower serum CC16 concentrations than women who did not. Bränn et al. (184) found that among 70 inflammatory markers, five were significantly elevated in women with PPD, including TNF ligand superfamily member (TRANCE), hepatocyte growth factor (HGF), IL-18, fibroblast growth factor 23 (FGF 23), and C-X-C motif chemokine 1 (CXCL1).

Mixed evidence has been found for the linkage between the C-reactive protein (CRP) and PPD. The review by Lambert and Gressier (185) suggested that the dosage of some inflammation biomarkers, including CRP, at the very end of pregnancy or immediately after delivery could predict PPD. Bränn et al. (186) found that the signal transducing adaptor molecule-binding protein (STAM-BP), axin-1, adenosine deaminase (ADA), sulfotransferase 1A1 (ST1A1), and IL-10 were lower in late pregnancy among women with PPD, and proposed a summary inflammation variable for predicting PPD. So far, further studies are needed to address inconsistencies in different results regarding the role of inflammatory processes in the development of PPD.

Overall, evidence for (anti-)proinflammatory cytokines as a diagnostic and predictive biomarker for PPD is mixed, which calls for more systematic studies in the future.

### Biomarkers From Biochemical Studies

Identification of nutritional and biochemical markers for PPD diagnosis has gained attention lately. Wójcik et al. (187) revealed a correlation between the severity of depressive symptoms and decreased serum zinc concentration third day after delivery in PPD patients. Roomruangwong et al. (188) found that lower serum zinc (and higher CRP) levels strongly predicted prenatal depression and physio-somatic symptoms, which all together predicted postnatal depressive symptoms.

The role of vitamin D in the development of depression has been studied in recent years, because it has regulatory functions in the immune system, and thus may act effectively as a neurosteroid. Christesen et al. (189) conducted a review on the impact of vitamin D on pregnancy, and found that a decreased vitamin D level during pregnancy may lead to PPD in one study and preeclampsia in several studies. In an exploratory study (190), a significant relationship over time was found between low 25-hydroxyvitamin D (25(OH)D) levels and high EPDS scores. Brandenbarg et al. (191) found that low early-pregnancy vitamin D status was associated with elevated depressive symptoms in pregnancy. Gur et al. (192) found that lower maternal 25(OH)D3 levels were associated with higher levels of PPD at all time points, and thus may be a factor affecting the development of PPD. Similarly, Robinson et al. (193) also reported that low vitamin D during pregnancy is a risk factor for the development of PPD symptoms. A cross-sectional study (194) found that higher dietary vitamin D intake was significantly associated with a lower prevalence of depressive symptoms during pregnancy. A recent prospective study found an association between lower prenatal log 25(OH)D and significantly more severe PPD symptoms, among women with higher levels of inflammatory markers (195). In a controlled study, Fu et al. (196) found an association between lower serum 25(OH)D levels measured 24 h after delivery and PPD. Fortunately, these findings seem to be in agreement with each other.

In a prospective cohort study of 238 pregnant women, Teofilo et al. (197) found that HDL-cholesterol concentrations were inversely associated with EPDS scores during pregnancy. In a prospective study of 266 Dutch women, Van Dam et al. (198) did not find an association between rapid serum cholesterol decline and the risk of developing PPD.

The PUFA status in late pregnancy was studied in a large sample of women, and only a weak link between PUFA in late pregnancy and PPD risk was reported (199). In a community-based prospective cohort, Markhus et al. (200) found that the DPA content, DHA content, ω-3 index, ω-3/ω-6 ratio, total PUFA score, and the ω-3 PUFA score were all inversely correlated with the EPDS score. Sallis et al. (201) found a weak positive correlation between ω-3 fatty acids and PPD. In another study, no association between plasma PUFAs and PPD was found (202).

For vitamin B12 and folate, a cross-sectional study reported no association between depressive symptoms and blood levels of vitamin B12 and folate (203). Lewis et al. (204) did not find strong evidence that folic acid supplementation can reduce the risk of depression during first 8 months of pregnancy. Another study found significantly higher homocysteine levels in women with PPD than in healthy controls, suggesting that the level of serum homocysteine might be a risk biomarker for PPD (205).

An increased kynurenine level has also been reported to be associated with the induction of depression. Depressive and anxiety symptoms in the early puerperium are associated with increased catabolism of tryptophan into kynurenine (206), and the increases in plasma kynurenine and the kynurenine/tryptophan (K/T) ratio were positively correlated with the anxiety and depression scores in the puerperium.

There have been some studies on gut microbiome as potential biomarkers recently (207). The coordination between the gut, the central nervous system, and the neuroendocrine and neuroimmune axes is referred to as the gut-brain axis (208). There are not many studies and thus not much has been known, regarding what role the gut-brain axis may play in the development of PPD. In a study of nearly 400 pregnant women (209), it was found that probiotic supplementation with *Lactobacillus* rhamnosus HN001 in pregnancy and postpartum period reduces the prevalence of PPD.

In summary, biochemical studies seem to suggest that low serum 25(OH)D levels during pregnancy may be a biomarker for PPD. Evidence for other biochemical markers for PPD is either mixed, very weak or negative, including levels of serum cholesterol, PUFA status, vitamin B12 and folate, kynurenine level, and gut microbiome.

### Omics-Based Biomarker Studies

Biomarker identification in neuropsychiatric disorders such as PPD and MDD can have important advantages and benefits, in terms of prediction and accurate diagnosis of a disease, and may provide more accurate and reliable information which can guide the selection and development of a cure or treatment. Gadad et al. (210) conducted a review on various omics approaches for identifying biomarkers of neuropsychiatric disorders. Many of the biomarkers mentioned in the review can be identified using multi-omics, which includes genomics, epigenomics, transcriptomics, proteomics, metabolomics, and lipidomics. These omics technologies have been actively applied in studies on MDD. See, e.g., the review by Sethi and Brietzke (211). Given the strong similarity between PPD and MDD, these omics technologies can in principle be quickly applied to the identification of biomarkers for PPD.

Metabolomics has recently been applied to unravel the serum metabolomic profile of PPD (212). Serum metabolomes of a group of women (*n* = 10) with PPD and a healthy control group (*n* = 10), all from Greece, were analyzed for targeted metabolomics using mass spectrometry. In the PPD group, increased levels of five metabolites were found, such as glutathione-disulfide, adenylosuccinate, and ATP. The data showed that molecular changes related to PPD were indeed detectable in peripheral material, and thus these changes may serve as diagnostic biomarkers.

A metabolomic profiling of morning urine samples of women with PPD, postpartum women without depression (PPWD), and healthy controls (HCs) was recently characterized using gas chromatography-mass spectroscopy (213). Twenty two (22) differential metabolites (14 up regulated and 8 down regulated) were found to separate PPD subjects from HCs and PPWD. Meanwhile, a panel of five potential biomarkers – formate, succinate, 1-methylhistidine, α-glucose and dimethylamine – was identified, which could be used to effectively distinguish PPD subjects from HCs and PPWD. Recently, using Liquid Chromatography Coupled to Quadrupole Time-of-Flight Mass Spectrometry, Zhang et al. (214) found that the urine metabolomic profiles of patients with PPD were different from those of HCs. Ten differentiating metabolites were found as main contributors to this difference.

So far, there have been far fewer studies on the identification of biomarkers for PPD than for MDD. Nevertheless, we expect that multi-omics technologies will be widely used in identifying biomarkers for PPD in future studies. Indeed, given the enormous advantages of the omics, they should be a major direction of PPD research in the future.

It should be cautioned that, due to possible partial overlap in genetic and biological risk factors and biomarkers among PPD, MDD and BD, a panel of multiple biomarkers may be needed to avoid misdiagnosis.

## Conclusions

PPD is a serious health issue for new mothers and has negative consequences on both the mothers and the children. Its high prevalence rate raises strong public health concerns. PPD is likely to be influenced by a multitude of risk factors, including biological, psychosocial, and even environmental factors. There have been various etiological models for PPD. However, no consensus has been reached so far. Typical treatments for PPD include psychotherapy and phamacotherapy, in the form of psychotherapy/counseling and antidepressant medications. While the US FDA has recently approved the first antidepressant medication for PPD, this medicine, Zulresso, has turned out to be extremely expensive, and thus is out of the reach for most PPD patients. This makes preventive measures more important, to protect one from developing PPD.

There has been increasing effort in the diagnosis of PPD using predictors, from both psychosocial and biological aspects. Furthermore, there are a variety of researches on PPD biomarkers so far, using different methods and approaches for characterizing and assessing PPD. While biomarker identification has shown a lot of promise for PPD research, nevertheless no biomarker is ready for clinical use as of today. From etiologic point of view, (epi)genetics and hormones may play a more fundamental role than biochemicals in the development of PPD. Nonetheless, biochemicals may as well be the right signatures or indicators that can be used for diagnosing and predicting PPD. Biomarkers in genetics and epigenetics may have a big potential for personal risk prediction. Several hormones, neurosteroids, and biochemicals have been identified in preliminary studies as potential biomarkers for predicting PPD, but further studies and substantiation are needed before they can be put into clinical use. So far, there are strong inconsistencies in various findings regarding predictors and biomarkers of PPD. These inconsistencies presumably have to do with the limited sample sizes, inconsistent depressive rating scales and timing for sampling, and inconsistent designs across different studies, as well as the high complexity of PPD. Further large-scale, integrative studies are needed to fully understand PPD. Given the objectiveness of biomarkers, we expect that the identification of biomarkers of PPD will be an important subject in future research. Despite that the application of multi-omics to the study of PPD has just begun recently, we strongly believe that modern multi-omics technologies will have a great potential in this arena.

While many studies have found associations or correlations between certain risk factors, predictors, or biomarkers and PPD, these correlations do not necessarily tell whether these risk factors, predictors, and markers are consequences or causes of PPD. A correct etiological model which is subject to comprehensive testing is crucial for uncovering the underlying cause of PPD and for developing the right medication and cure for PPD.

Finally, we end this review by presenting a brief speculative model for the etiology of PPD, based on the findings summarized hereinabove. The hormonal withdrawal theory has largely been proved to be wrong, as the hormonal withdrawal is a normal process every pregnant woman has to undergo upon parturition. This is a desired and necessary biological change that is adapted to pregnancy and parturition, as a result of human evolution. However, for a portion of women the biological system may not perform as perfectly as expected, due to the high complexity of human body and a wide range of genetic and epigenetic variations, as well as environmental, psychosocial and biological factors. In other words, for those the body does not fulfill and cope with the hormonal withdrawal in a perfect manner, depressive disorders to various degrees may develop. Thus, the ultimate goal of studying various predictors and biomarkers for PPD will be to catch such an imperfection at an early stage so that it can be remedied or prevented in time.

## Author Contributions

YY completed the literature survey and manuscript writing. J-CL initiated and oversaw the project. H-FL, JC, Z-BL, Y-SH, and J-XC participated in the discussion and the manuscript preparation. All authors contributed to the article and approved the submitted version.

## Conflict of Interest

The authors declare that the research was conducted in the absence of any commercial or financial relationships that could be construed as a potential conflict of interest.
